# Human pre-B cell receptor signal transduction: evidence for distinct roles of PI3kinase and MAP-kinase signalling pathways

**DOI:** 10.1002/iid3.4

**Published:** 2013-10-30

**Authors:** Kolandaswamy Anbazhagan, Amrathlal Rabbind Singh, Piec Isabelle, Ibata Stella, Alleaume-De Martel Céline, Eliane Bissac, Brassart Bertrand, Nyga Rémy, Taylor Naomi, Fuentes Vincent, Jacques Rochette, Kaïss Lassoued

**Affiliations:** 1 Inserm/UMR925, Université Picardie Jules Verne, Laboratoire d'Immunologie, UFR de Médecine3, rue des Louvels, 80036, Amiens, France; 2 CNRS/UMR 5535, Institut de Génétique Moléculaire de Montpellier1919 Route de Mende, 34293, Montpellier, Cedex 5, France

**Keywords:** MAPK, PI3K, pre-B cells, pre-BCR, receptor signalling

## Abstract

Pre-BCR acts as a critical checkpoint in B cell development. However, its signalling cascade still remains indistinctly characterised in human. We investigated pre-BCR signalling pathway to examine its regulation in normal primary pre-B lymphocytes and pre-B cell lines. In cell lines, early signalling events occurring after pre-BCR stimulation include phosphorylation of Lyn, Blk and Syk together with ZAP70, Btk, Vav, PLC-γ2 and various adaptor proteins, such as BLNK, LAB, LAT and SLP-76. Further downstream, these molecules induced activation of the PI3K/AKT and MAP-kinase resulting in an augmentation of canonical NF-κB pathways and cFos/AP1 activation. PI3K and MAPK exerted opposing effects on the pre-BCR-induced activation of the canonical NF-κB and c-Fos/AP1 pathways. Immediate nuclear export of FoxO3A and delayed import of IRF4 were additional events observed after pre-BCR crosslinking in primary cells. Pre-BCR-induced down-regulation of *Rag1*, *Rag2*, *E2A* and *Pax5* transcripts occurred in a PI3K-dependent manner. Finally we bring evidence that pre-BCR stimulation or co stimulation with CD19 enhances cell cycle signal.

## Introduction

The dynamic process of B-cell differentiation requires co-ordination of various receptors, signalling molecules, transcription factors and the recombinases. The surface expression of pre-B cell receptor (pre-BCR) complex, which is composed of μHC, the surrogate light chain (SLC) and the Igα/Igβ transducing complex, appears at the end of pro-B/pre-B I stage [1–3]. This receptor constitutes a check point in early steps of B cell development [4]. There is significant evidence suggesting the role of pre-BCR in the pathogenesis of lymphoid malignancies [5]. Therefore, dissection of the molecular mechanisms through which this receptor exerts its function is important for understanding the regulation of B lymphopoiesis and its implication in the neoplastic transformation of precursor B cells.

During the early stages of B-cell development, a shift between the cell cycle and DNA rearrangement is a crucial event to keep the differentiation process under control. This shift between the cellular events is regulated by the signals exerted by pre-BCR to control the clonal expansion of pre-B cells and the rearrangement of immunoglobulin light chain (IgLC) [6,7]. The regulation is achieved by two distinct signalling states at varied threshold levels from the pre-BCR. The interchange between two distinct functions of pre-BCR signalling is coordinated by the downstream molecule, BLNK (SLP65) [8,9]. BLNK regulates MAPK and PI3K-AKT pathways, which are involved in two distinct signalling events promoting light chain gene rearrangement and cell cycle, respectively [7,10].

In mice, studies have shown association of IL7-receptor (IL7R) in promoting the cell cycle in pre-B cells via PI3K-AKT pathway [11–13]. By inhibiting FoxO activity, AKT negatively regulates the expression of *Rag* and BLNK, therefore promoting cell cycle events. Pre-BCR plays an important role of attenuating IL7R function, thereby promoting IgLC rearrangement and differentiation of pre-B cells, which is attained by pre-BCR-induced signalling via Syk-BLNK to inhibit PI3K/AKT pathway. This phenomenon also activates FoxO and *Pax5* transcription factors, thereby up-regulating expression of *Rag1/2* and IRF4 to promote IgLC rearrangements.

On contrary to mice, human pre-B cells do not react over IL7 stimulation for promoting cell cycle. Normal development of B-cells in severe combined immunodeficiency (SCID) patient, with mutation in IL7R gene has been the basis for the argument that human B-cell development is IL7 independent [14,15]. Johnson et al. [16] proposed that IL7 does not activate PI3K/AKT pathway in normal human pre-B cells [16]. On the other hand, the pre-BCR is involved in dual function to control the switch between the signals for the cell cycle and the IgLC gene rearrangement. Since BLNK adaptor molecule is a part of this transitional switch, its activation and inactivation may involve feedback mechanisms to regulate MAPK and PI3K/AKT pathways [7]. Further downstream, regulatory roles of MAPK and PI3K/AKT on transcription factors (NF-κB, *Pax5*, *E2A*) are less studied, although GADD45a-mediated FoxO1-dependent expression of *Rag1* transcript and their negative regulation by the PI3K/AKT is demonstrated in mice [17]. The central question of how these two pathways regulate the downstream transcription factors and their targets still remains unanswered in human. Existence of any cross talk between these two pathways in controlling the threshold of pre-BCR signalling strength has to be investigated. In this study, we describe the pre-BCR-associated signalling pathways using human pre-B cell lines and normal primary pre-B cells. We provide evidence that pre-BCR-induced activation of PI3K and MAPK is SYK and SRC-dependent. Furthermore we also show that pre-BCR exerts dual effect to regulate NF-κB and c-Fos activation via PI3K and MAPK. The results in the present work provide evidence that pre-BCR down-modulates *Rag1*, *Rag2*, *Pax5* and *E2A* expression in a PI3K-dependent manner. This receptor induces early activation of c-Cbl as well, which has the potential to negatively regulate SYK, SRC kinases and AKT. Altogether, our finding indicates that MAPK and PI3K play an important regulatory role among the series of checkpoints in the pre-BCR signalling cascade.

## Results

### Early pre-BCR signaling molecules activate PI3K/AKT and Ras/MAPK pathways

To study the effect of pre-BCR stimulation we initially used two pre-B cell lines, 697 and Nalm6. After examining the quality of cell lines by checking their surface markers (Fig. S1A), pre-BCR was stimulated by using anti-μ F(ab')2 antibody. Pre-BCR crosslinking resulted in enhanced proliferation and increased S-phase of pre-B cell line – 697 (Fig. S2A and B). This proliferation was associated with increased phosphorylation of cell cycle related proteins, p21 and Rb, in addition to overexpression of p27 and Myc (Fig. S2C and D). On investigating the downstream signalosome, pre-BCR cross-linking rapidly induced phosphorylation of LYN, SYK, BLNK, Vav, Btk and PLC-γ2 (Fig. S3A), in addition to Igα (not shown), as previously reported [18]. This also induced phosphorylation of Blk, but not other members of the Src kinase family including Fyn, Hck, Fgr and Shc. Furthermore, LAT, LAB, SLP76 and Zap70 adaptor molecules were found to be expressed in the cell line and were phosphorylated rapidly following pre-BCR stimulation (Fig. S3A and B).

As pre-BCR stimulation promotes the recruitment of p85-PI3K regulatory subunit and phosphorylation of AKT [18–20], we examined the cell lines for the vitality of the PI3K and MAPK pathway by stimulating pre-BCR for various time points and monitored their phosphorylation level. Here, we show that pre-BCR stimulation results in the rapid phosphorylation of AKT (Ser473) in both Nalm6 and 697 cells (Fig. S3C). Furthermore, phosphorylation of the two AKT substrates, GSK3β and FoxO3A (FKHRL-1), was significantly enhanced upon pre-BCR stimulation within 5 and 15 min, respectively (Figs. S3C and 3A). Although pre-BCR stimulation results in phosphorylation of ERK1/2, its ability to induce phosphorylation of p38, the other arm of MAPK pathway has been debated [12,21]. We observed that human pre-BCR promoted phosphorylation of ERK1/2 together with p38 MAPK, but not JNK. The receptor stimulation also promoted the activation of Ras, an upstream signalling molecule in the MAPK pathway (Fig. S3D) [19].

Stimulation of pre-BCR also induced rapid phosphorylation of c-Cbl on Y731 and Y774 residues in both cell lines within 30 s after stimulation (Fig. S4A and B). Additionally, ZAP70 co-precipitated with c-Cbl upon pre-BCR stimulation, suggesting physical association of these two molecules (Fig. S4C). No significant changes in phosphorylation of SHP1, SHP2 or SHIP were observed following pre-BCR stimulation.

### SYK is an upstream regulator in controlling PI3K/AKT and MAPK pathways

**Figure 1:**
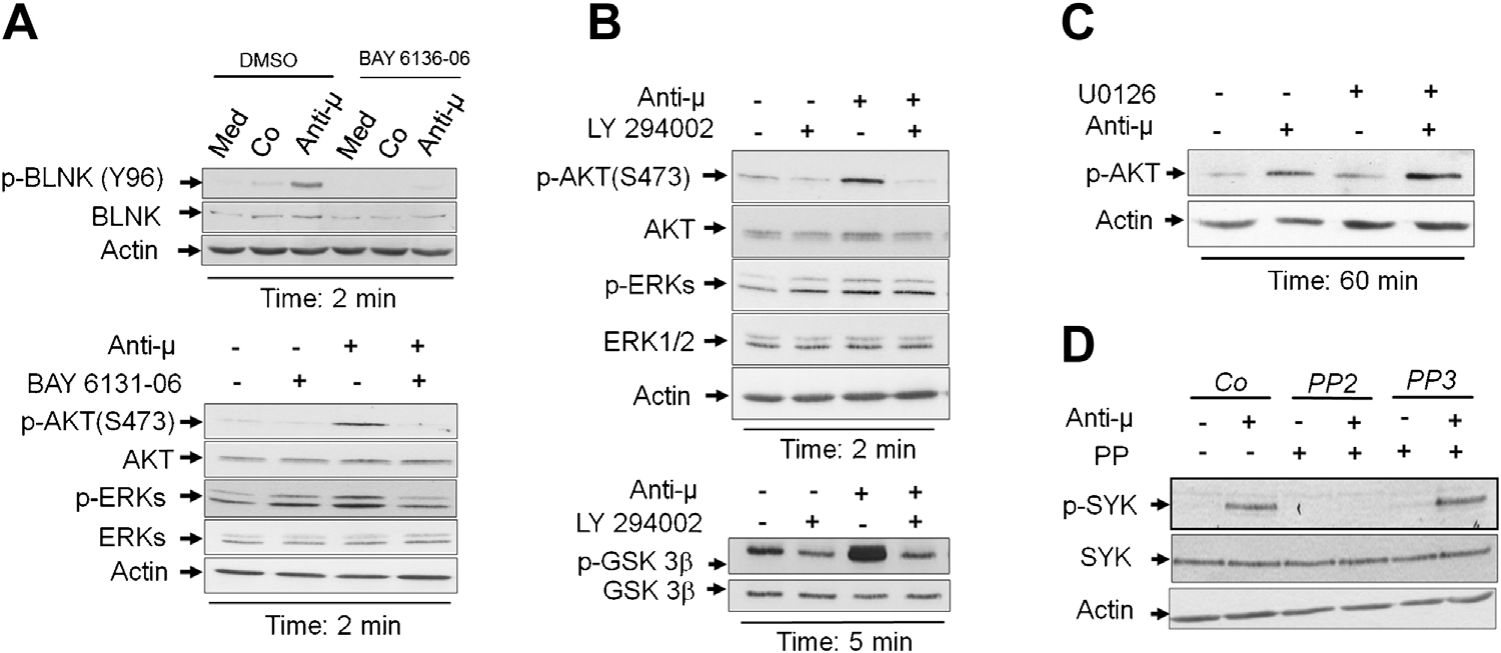
Ordering of SRC, SYK, AKT and MAPK in pre-BCR signalling cascade. Pre-BCR was cross-linked using anti-µHC F(ab')2 antibody in presence or absence of BAY 6136-06 (SYK inhibitor) (A), Ly294002 (PI3K/AKT inhibitor) (B), U0126 (MEK1/2 inhibitor) (C), PP2 (SRC inhibitor) or PP3 (inactive homolog of PP2) (D). Total cell lysates were analysed by western blotting for p-BLNK, p-AKT, p-ERK, p-GSK3β or p-SYK. The blots were re-probed for non-phosphorylated forms of all signalling molecules and actin as loading control. Data represented is one of two independent experiments. Med: medium only (RPMI 1640).

**Figure 2:**
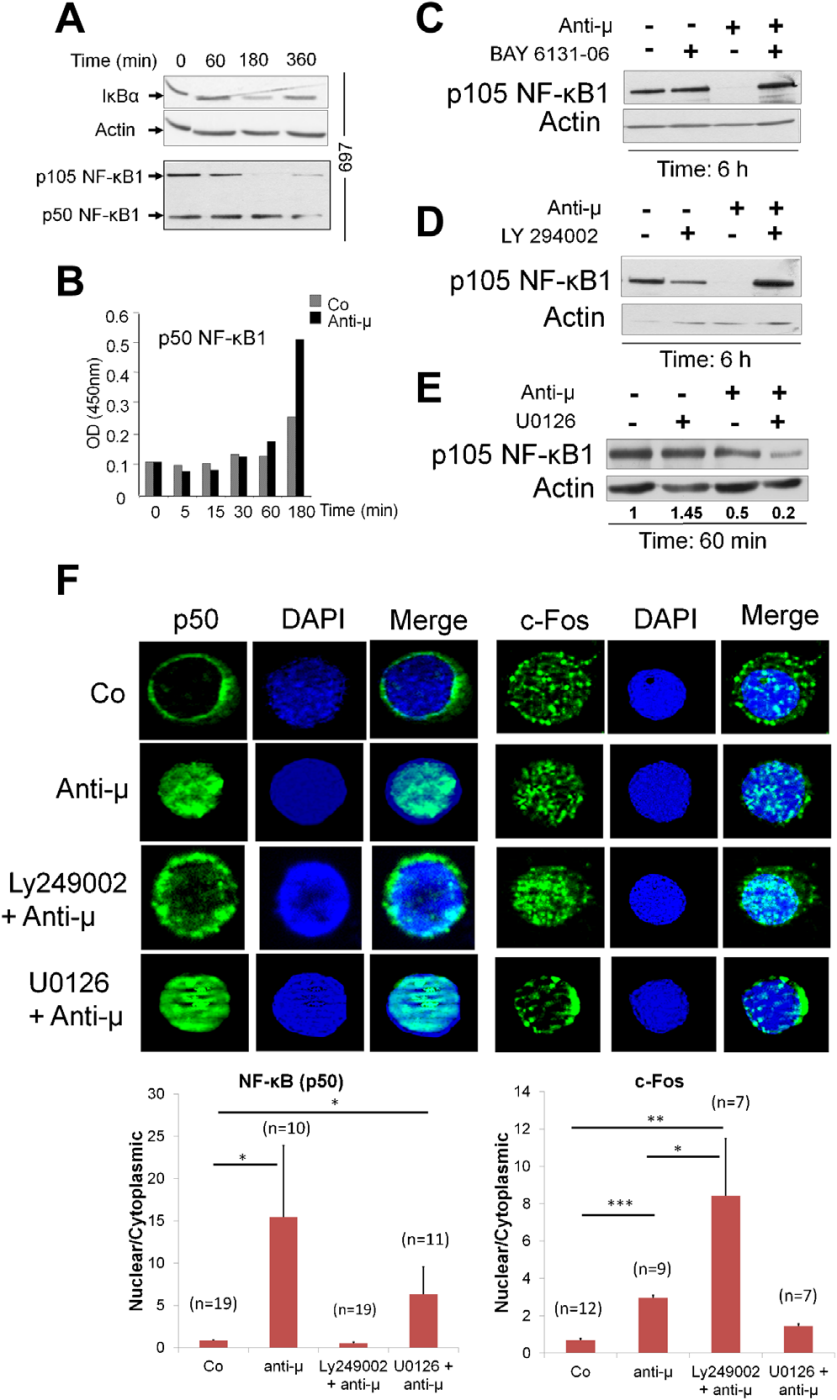
Pre-BCR-induced activation of NF-κB and c-Fos and their regulation by PI3K and MAPK. (A) Serum starved 697 cells were cross-linked with anti-µHC or control F(ab')2 antibodies. Cell lysates were prepared and blotted with specific antibodies to detect IκBα, p105 and p50. The figure is representation of two independent experiments. Actin detection was used as a loading control. (B) Nuclear translocation of transcription factors p50 and their binding to oligonucleotide containing specific consensus motif were detected using the TransAM™ ELISA-based kit (Active Motif, Carlsbad, CA, USA). Results are represented as a mean value of optical density (OD) derived from two independent experiments. (C–E) Pre-BCR was cross-linked using anti-µHC F(ab')2 antibody with or without BAY 6136-06 (SYK inhibitor) or Ly294002 (PI3K/AKT inhibitor) or U0126 (MEK1/2 inhibitor) and were analysed by western blotting for p105- NF-κB. Actin was probed as loading control. Data represented is one of two independent experiments. (F) Role of PI3K and MAPK in pre-BCR-induced NF-κB and c-Fos translocation in human primary pre-B cells: Normal pre-B cells from bone marrow was sorted, incubated with anti-µHC or control F(ab')2 with or without LY294002 or U0126 for 3 h. Immunofluorescence staining was performed to examine the nuclear translocation of NF-κB (p50) and c-Fos. DAPI was used as a nuclear stain. The lower panel shows the histogram plotted based on the quantification of nuclear/cytoplasmic ratio of NF-κB (p50) (left) and c-Fos (right). Data are derived from 7 to 18 pre-B cells (n) under each condition and presented as a mean value ± SEM. Immunofluorescence image represents one of two independent experiments. *P ≤ 0.05; **P ≤ 0.01; ***P ≤ 0.001.

In order to determine the roles of SYK, PI3K and MAPK in pre-BCR signalling, cells were treated with their respective pharmacological inhibitors followed by stimulation with anti-μ F(ab')2 antibodies. Use of BAY61-3606 (SYK inhibitor) resulted in complete inhibition of pre-BCR-induced BLNK, AKT and ERK1/2 phosphorylation (Fig. 1A), p105 NF-κB1 degradation (Fig. 2A and C) and subsequent abrogation of cell growth (not shown). LY294002 (PI3K inhibitor) prevented AKT and GSK3β phosphorylation, but did not modify ERK1/2 phosphorylation (Fig. 1B). PI3K inhibition also prevented p105 NF-κB1 degradation indicating that pre-BCR-mediated NF-κB activation is PI3K-dependent (Fig. 2D). In contrast, treatment of pre-B cells with the U0126 (MEK1/2 inhibitor) resulted in the enhancement of the pre-BCR-induced NF-κB activation. This is shown by an earlier and enhanced degradation of p105 NF-κB without affecting AKT phosphorylation, indicating that negative role is played by the MAPK pathway in preventing NF-κB activation (Figs. 2E and 1C). Consequences of AKT or ERK knock-down by means of shRNA could not be analysed since electroporation of their respective or control shRNA plasmids resulted in high cell mortality.

To determine the role of the Src kinases in this signalling cascade, cells were treated with the Src inhibitors (PP1 and PP2) and with an inactive analogue of PP2 (PP3) as a control. We demonstrated that PP1 (data not shown) and PP2, but not PP3 prevented pre-BCR-induced SYK phosphorylation (Fig. 1D). Reproducible results were obtained with another Src inhibitor, SU-6656 (data not shown). Altogether these results indicate that Src kinases act upstream SYK, which in turn activates both MAPK and PI3K/AKT. MAPK is also shown to negatively regulate the AKT-dependent pre-BCR-induced NF-κB1 activation.

### PI3K/AKT and MAPK pathways regulate NF-kB activation

Pre-BCR-induced NF-κB activation is considered to be important for the survival of pre-B cells [22–24]. However, direct evidence on the link between pre-BCR and the NF-κB activation remains unknown. We observed that pre-BCR stimulation resulted in decreased IκBα expression and p105 (NF-κB1 precursor) degradation, indicating an activation of the NF-κB canonical pathway (Fig. 2A). NF-κB activation started after 1 h of stimulation, reaching a maximum between 3 and 6 h. Using ELISA-based method (TransAM™), we show that this process was associated with nuclear translocation of p50 NF-κB1 and its binding to an oligonucleotide containing a specific consensus sequence (Fig. 2B). In contrast, there was no activation of NFATc1, STAT1, STAT3 or STAT5 after pre-BCR stimulation. Using pharmacological inhibitors, we show that SYK (BAY6131-06) and PI3K (LY294002) prevented degradation of p105-NF-kB upon pre-BCR stimulation, but not by the MEK inhibitor (U0126; Fig. 2C–E). These results noticeably show that pre-BCR is directly linked to NF-kB activation via PI3K. Effects of pre-BCR stimulation on c-Fos/JunB (AP1) and FoxO3A could not be analysed using immunofluorescence study due to their high baseline activation in both Nalm6 and 697 pre-B cell lines.

**Figure 3:**
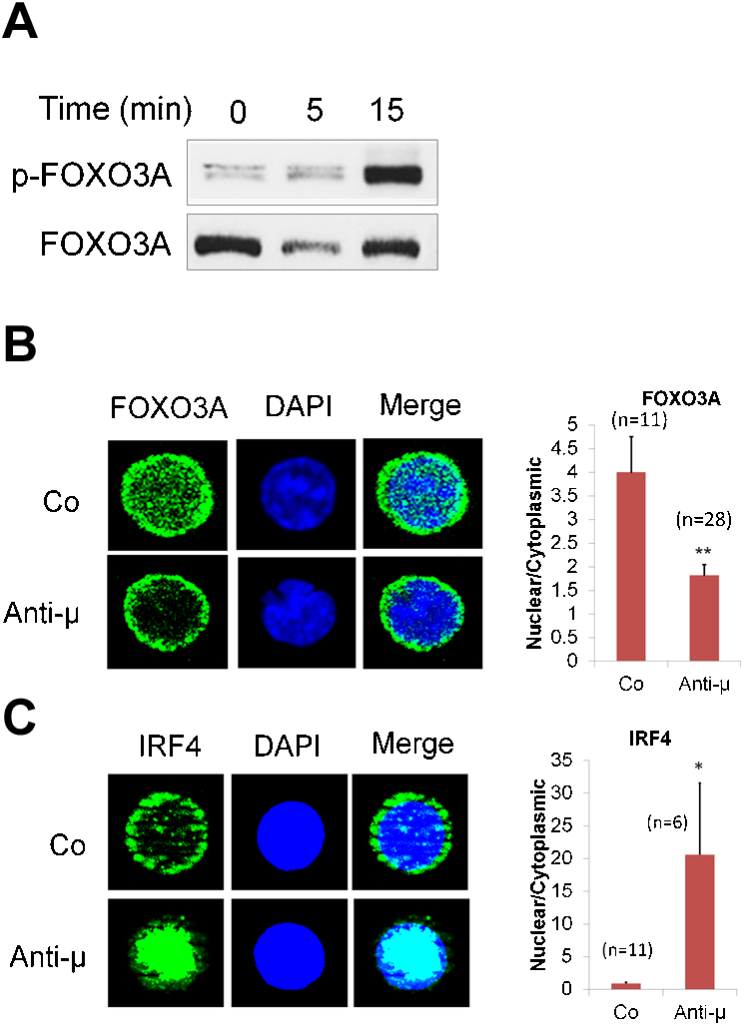
Nuclear translocation of FOXO3A and IRF4. (A) Serum starved Nalm6 were treated with anti-µHC or control F(ab')_2_ antibody and analysed by western blotting for FOXO3A (FKHRL-1) phosphorylation. (B and C) Primary bone marrow pre-B cells were treated with anti-µHC or control F(ab')_2_ antibodies for 3 h (FoxO3A) or 24 h (IRF4) in independent experiments and immuno-stained with rabbit anti-FOXO3A or anti-IRF4 followed by goat anti-rabbit Ax488. DAPI was used as a nuclear stain. Panel to the right of figures B and C shows histogram corresponding to quantification of nuclear/cytoplasmic ratio of FoxO3A and IRF4. Data are derived from 6 to 28 pre-B cells (*n*) under each condition and presented as a mean value ± SEM. Immunofluorescence image represents one of two independent experiments derived from biological replicates. *P ≤ 0.05; **P ≤ 0.01.

When examined in bone marrow primary pre-B cell, immunofluorescence experiment revealed nuclear translocation of p50 NF-κB after 3 h of pre-BCR stimulation (Fig. 2F). Stimulation of pre-BCR also resulted in an enhancement of c-Fos nuclear translocation associated with decreased cytoplasmic location (Fig. 2F). Treatment of normal pre-B cells with BAY61-3606 resulted in complete apoptosis (100% dead cells at 48 h), whereas treatment with LY294002 or U0126 did not alter cell survival significantly even after 24 h, which was also shown in our published data before [25]. LY294002 prevented pre-BCR-induced p50 NF-κB nuclear translocation whereas U0126 did not. Conversely, c-Fos nuclear translocation was inhibited by U0126-treatment and slightly but consistently enhanced by LY294002-treatment in association with a decrease in its cytoplasmic location. In addition to NF-κB, pre-BCR stimulation also resulted in nuclear export of FoxO3A within 3 h and nuclear import of IRF4 only after 24 h (Fig. 3).

### Opposing effect of PI3K/AKT and MAPK pathways on *Rag1/2, E2A* and *Pax5* expression

**Figure 4:**
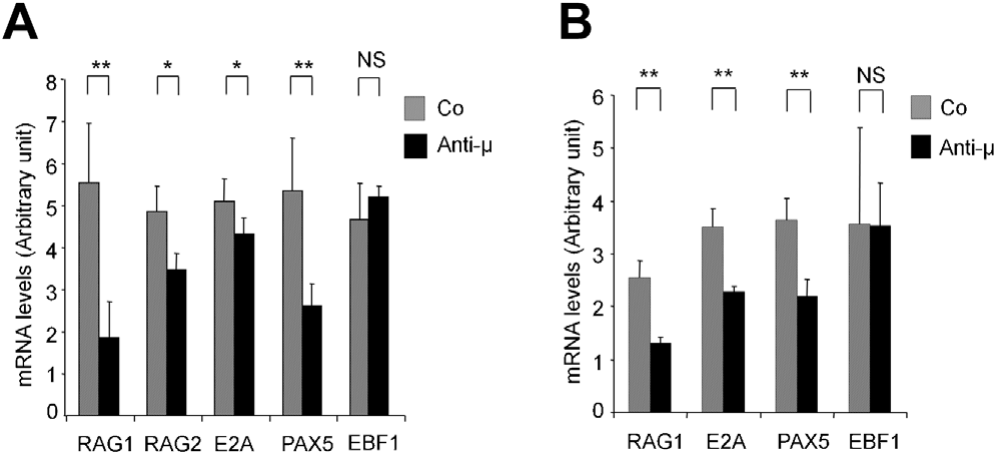
Pre-BCR-dependent down regulation of *Rag1/2*, *E2A* and *Pax5* expression in cell line and primary normal pre-B cells. (A) Serum starved 697 cells were treated with anti-µHC or control (Co) F(ab')2 for 24 h and examined for the expression of *Rag1*, *Rag2*, *E2A*, *Pax5* and *EBF1* using Taqman based Real-Time PCR. (B) Normal primary pre-B cells were sorted from human bone marrow, stimulated with anti-µHC or control F(ab')2 antibodies. Expression of *Rag1*, *E2A*, *Pax5* and *EBF1* mRNA was quantified by Taqman-based Real-Time PCR as a ratio to 18S mRNA level. Data sets shown here were derived from five biological replicates from unrelated samples and are represented as mean ± SD (*P < 0.05, **P < 0.01 and NS = no significant).

In mice, expression of *Rag1/2* genes is down-regulated after the rearrangement of the heavy chain in pre-B cells [26]. This down-regulation of *Rag1/2* expression is mediated by the pre-BCR signalling via AKT pathway, which inhibits the GADD45a/MEKK4-mediated FOXO1 activation [17,27]. However its regulation in human pre-B cells is not studied extensively and always found to be extrapolated from the mouse counterpart. Using quantitative RT-PCR, we show that pre-BCR stimulation resulted in a significant down-regulation of *Rag1* and *Rag2* expression (−66% *P* < 0.01 and −28%, *P* < 0.05, respectively; Fig. 4A). This effect was evident after 24 h of pre-BCR stimulation and was transient (*Rag1/2* transcripts returned to baseline levels after 48 h). This was accompanied with a significant down-regulation of *Pax5* (−51%, *P* < 0.01) and to a lesser extent of *E2A* (−15%, *P* < 0.05; Fig. 4A) but not of *EBF1*. Besides *Pax5* and *E2A*, we did not observe any changes in the mRNA levels of various other transcription factors that are reported to regulate *Rag1* and *Rag2* transcription, namely FoxO1, FoxO3, FoxO4, Myb, MAZ, LEF1 and SP1 (results not shown). Similar results were obtained in human normal pre-B cells with the down-regulation of *Rag1* (−48%, *P* < 0.01), *Pax5* (−40%, *P* < 0.01) and *E2A* (−35%, *P*< 0.01) transcripts, while *EBF1* expression remained unchanged (Fig. 4B).

**Figure 5:**
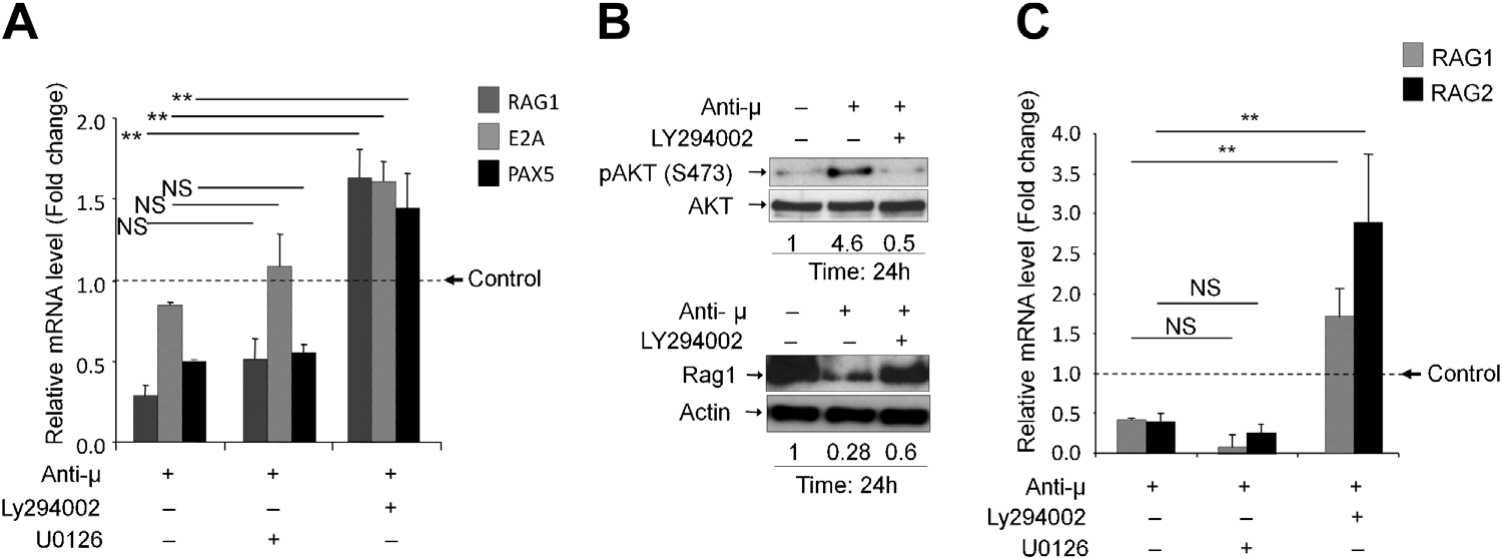
Role of MAPK and PI3K/AKT in pre-BCR-dependent *Rag1/2*, *Pax5* and *E2A*. (A) 697 cells were stimulated using anti-µHC with or without Ly294002 or U0126 for 24 h and examined for the change in the expression level of *Rag1*, *E2A* and *Pax5* with respect to endogenous control, 18S rRNA level. Dotted line refers to the relative mRNA expression in control cells (treated with isotype control F(ab')_2_ antibody). Data shown was derived from four independent experiments and presented as mean ± SD (**P < 0.01 and NS = no significant). (B) 697 cells cells pre-treated with or without Ly294002, were cross-linked with anti-µHC F(ab')_2_ or control F(ab')_2_ antibodies for 24 h in presence of 2% FCS and blotted to quantify RAG1 protein. The bands were quantified using imageJ. The values reported below the figure correspond to fold change. Data represented is one of two independent experiments. (C) Normal pre-B cells were stimulated using anti-µHC with or without LY294002 or U0126. The expression level of *Rag1* and *Rag2* mRNAs level was evaluated by RT-PCR as a ratio to 18S mRNA expression and the differences were compared using Student's *t*-test (**P < 0.01). Dotted line refers to the relative mRNA expression in control cells (treated with isotype control F(ab')_2_ antibody). Data shown was derived from four biological replicates from unrelated samples and are represented as mean ± SD (**P < 0.01 and NS = no significant).

In presence of LY294002, pre-BCR stimulation induced an up-regulation of *Rag1* (+470%, *P* < 0.01), *E2A* (+90%, *P* < 0.01) and *Pax5* (+190%, *P* < 0.01) transcripts (Fig. 5A). Whereas, effects of pre-BCR stimulation on the expression of the *Rag1* gene and the transcription factors did not change in U0126 pre-treated cells. When examined for the *RAG1* at protein level, pre-BCR cross-linking induced a sharp decrease in RAG1 protein expression at 24 h. This down-regulation was still observed in U0126-treated 697 cells (not shown) but was partially prevented in LY294002 treated cells (Fig. 5B). In LY294002-treated primary cells, *Rag1* and *Rag2* gene expression was up regulated (+130%, *P* < 0.01 and +251%, *P* < 0.01, respectively) following pre-BCR crosslinking, whereas in the presence of U0126 the pre-BCR-induced *Rag1*/*Rag2* down-modulation remained unchanged (Fig. 5C).

## Discussion

This work has been performed using two different pre-B cell lines and normal B cell precursor. We have analysed the pre-BCR-induced activation of a large spectrum of kinases and adaptors and regulation of its signalling. We demonstrate some of the main events occurring up on stimulation of pre-BCR in human cell lines and primary pre-B cells: (i) the downstream of pre-BCR signalling is a SRC-dependent event, (ii) pre-BCR-induced activation of NF-κB, which promotes cell survival and receptor editing, is a PI3K-AKT-dependent process, (iii) FOXO3A inactivation, possibly by AKT, resulted in export from the nucleus and (iv) Pre-BCR-induced activation of NF-κB and FOXO3A inactivation resulted in down-regulation of *Rag1/2*, *Pax5* and *E2A*, which were distinctly regulated by the PI3K/AKT and MAPK pathways.

Pre-BCR is a major signalling event in pre-B cell, which exerts dual function in promoting cell proliferation and differentiation [7]. Previous reports have shown that pre-BCR stimulation results in phosphorylation of various cytosolic molecules [4,6,28]. In agreement with the current model proposed for pre-BCR signalling cascade, we show that the earliest events associated with human pre-BCR stimulation include phosphorylation of LYN, BLK, SYK, BLNK, Btk, Vav and PLC-γ2 together with activation of the PI3K/AKT pathway and the ERK1/2 and p38 arms of the Ras/MAPK pathway. SRC and SYK are known to play a crucial role in B lymphopoiesis [29,30]. It is evident from our study that the inhibition of SYK results in abrogation of pre-BCR signalling and hampers survival of normal human pre-B cells in ex vivo condition. We also show that pre-BCR triggers phosphorylation of ZAP70, another member of SYK family, though less efficiently than SYK. This finding is in line with the minor role played by ZAP-70 in the earliest steps of mouse B lymphopoiesis and with the normal B cell development in ZAP70 deficient SCID patients [31,32]. However whether ZAP-70 can compensate loss of SYK in humans, as reported in mouse needs to be established [33].

**Figure 6:**
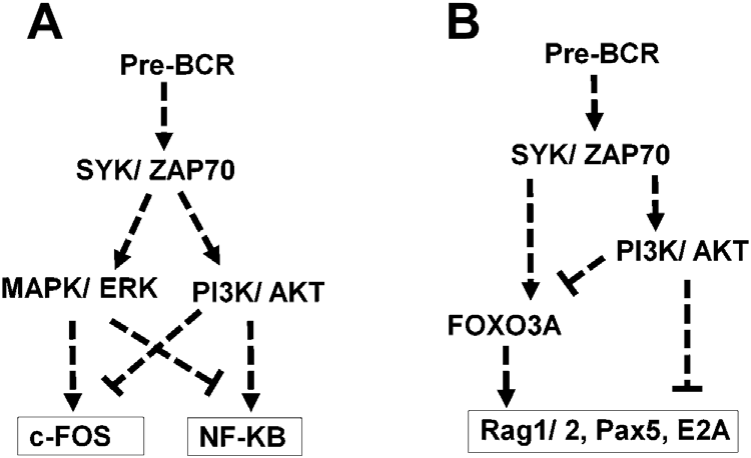
A model of human pre-BCR-induced signal transduction depicted from the current study. (A) The stimulation of pre-BCR triggers activation of SYK/ZAP70 tyrosine kinases which in turn leads to activation of MAPK/ERK and PI3K/AKT pathways. MAPK and PI3K pathway show opposing effect in order to maintain the optimal activity of c-Fos and NF-κB, which may control the normal development of pre-B cells. (B) The pre-BCR-induced down-regulation of *Rag1/2* expression is PI3K-dependent and involves FOXO3A.

Various transcription factors are involved in pre-BCR function [34,35]. NF-κB is one of the key factors involved in regulation of survival, cell proliferation and differentiation at different stages of B-cell development [22,30,36]. We show that, in either cell lines or normal primary pre-B cells, pre-BCR activates the canonical NF-κB, which is previously shown to be involved in inducing expression of anti-apoptotic protein family members to extend the survival and promote proliferation of immature-B cells [22,37]. We also provide evidence in normal pre-B cells that pre-BCR stimulation rapidly activates c-Fos, a member of the AP1 family that has the potential to promote cell proliferation, differentiation and survival [38]. We also demonstrate that PI3K/AKT and MAPK exert opposing effects on pre-BCR-induced NF-κB and c-Fos activation as depicted in the diagram (Fig. 6). Activation of the PI3K, MAPK, NF-κB and c-Fos/AP1 pathways are likely involved in the pre-BCR-induced cell cycle entry that is shown to be associated with changes in the levels of phosphorylation or expression of p21^cip1^, p27^kip1^, Rb and Myc (Fig. S2). AKT-induced FoxO3A phosphorylation and subsequent nuclear exportation might also be implicated in this process [7]. CD19, a receptor that was also reported to potentiate pre-BCR function, is shown here to converge with pre-BCR signalling at ERK1/2 and AKT as well as at the downstream cell cycle regulatory factors such as p27^kip1^ and Rb (Fig. S2C and F).

It has been suggested that pre-BCR stimulation in mice down-modulates expression of *Rag* transcripts, a process thought to be associated with IgHC allelic exclusion while protecting the pre-B cells from *Rag*-associated genomic instability and cell transformation. *Rag* down-modulation in mouse large pre-B cells is a SYK-dependent process involving PI3K/AKT pathway and FoxO transcription factors [9,17]. However, the link between these regulatory mechanisms to the pre-BCR signalling has not been experimentally demonstrated so far. Using either human cell lines or normal primary pre-B cells, we conclude that pre-BCR-induced down-regulation of *Rag1/2*, *Pax5* and *E2A* is an AKT-dependent, but not a MAPK-dependent. RAG1 protein is also down-regulated in a PI3-K/AKT-dependent manner at a much higher extent than the transcripts, probably as a consequence of the inhibition of FoxO3A and p27^kip1^ which has the potential to stabilise or degradation of RAG1 protein upon its phosphorylation [7,17,39,40]. Additionally, up-regulation of *Rag1/2*, *E2A* and *Pax5* following pre-BCR stimulation in the presence of PI3K inhibitor indicates that PI3K/AKT negatively regulates these molecules. This suggests either a negative feedback regulatory loop at the AKT level or activation of an unidentified pre-BCR dependent signalling pathway that attenuates the pre-BCR-mediated AKT-dependent down-regulation of *Rag1/2*, *Pax5* and *E2A*.

Nuclear export of FOXO3A, possibly due to their phosphorylation by AKT occurred within 3 h indicating that cells were influenced to undergo cell cycle. This is in line with the literature stating that B-cell proliferation is dependent on PI3K-mediated inactivation of FOXO transcription factors [41]. On other hand, pre-BCR-induced nuclear import of IRF4 after 24 h suggested that cells were inclined towards differentiation.

Evidences from literature point out on the importance of PI3K/AKT in regulation of cell cycle and IgL rearrangement [9,42,43]. PI3K-dependent down-regulation of *Rag1*-GFP reporter gene or promotion of artificial recombination up on inhibition of PI3K in B cells, suggests that PI3K promotes cell cycle by suppressing the recombination machinery [43]. In agreement with these reports, we show that pre-BCR-mediated PI3K/AKT activation supports the cell survival and cell cycle events by activating NFκ-B (Fig. 2), inhibiting FoxO3A (Fig. 3) and suppressing *Rag1/2*, *Pax5* and *E2A* expression (Fig. 5).

This study presents one of the fewer investigations of human pre-BCR signalling cascade using cell lines as well as human primary normal pre-B lymphocytes. Pre-BCR signalling is modulated by co-stimulatory signals and appears to be tightly self-controlled with the ability to regulate its own expression and functions through BLNK adaptor, IRF-4, 8 [34,44,45], and probably also via *Pax5* and *E2A* as suggested here. This receptor can also limit its own ability to activate NF-κB and c-Fos via MAPK and to down-regulate *Rag1/2*, *E2A* and *Pax5* through self-controlled AKT-dependent mechanism. Further studies would eventually be helpful in explaining how homotypic interaction, ligand-independent and ligand-dependent (Galectin-1, haparan sulphate or post-translational modifications of the receptor) pre-BCR signalling differ in regulation of downstream signalling [45–49]. From the known data, we hypothesise that deregulation of the pre-BCR signalling pathways may result in an uncontrolled pre-B cell proliferation, alteration in pre-B cell differentiation and increased genetic instability due to persistent or increased *Rag* genes expression. Characterizing the molecular defects of pre-BCR signalling in pre-B ALL may also shed light onto the link between the transformation process and the abnormal blockade of B cell development.

## Materials and Methods

### Cell culture and specific cell treatments

Nalm6 and 697 human pre-B cells were cultured as previously described [1,25]. Prior to each experiment the cell lines were examined for their surface markers (Fig. S1A). In all experiments, cells were grown for 24 h and then depleted of serum for at least 2 h. Cells were treated with anti-μ F(ab')_2_ or control F(ab')_2_ antibodies (20 µg/ml) for the indicated length of time. In some experiments, cells were incubated with 10 µM of the LY294002 (PI3K/AKT inhibitor) or with 1 µM of the BAY 6136-06 (SYK inhibitor) or the U0126 (MEK1/2 inhibitor) for at least 30 min before pre-BCR stimulation. For cell cycle analysis, ethanol fixed cells were treated with RNase and stained using propidium iodide followed by flow cytometer analyses. The percentage of living cells was evaluated using the trypan blue dye exclusion assay.

### Cell lysates preparation and western blotting

Cells were grown in 75 cm^2^ flasks, serum starved for 2 h, and treated with various agents as indicated above. Cells were then washed with ice-cold PBS and treated for 20 min on ice in lysis buffer (NP40) containing protease inhibitors and phosphatase inhibitors. Lysates were clarified by centrifugation at 14,000*g* for 20 min at 4 °C. In some experiments cells were lysed in SDS sample buffer to obtain total cell lysate. Proteins (50 or 100 µg) were separated on SDS-polyacrylamide gels and transferred onto nitrocellulose membranes. Phosphorylation of kinases was analysed by immuno-blotting using antibodies specific to the phosphorylated form of each kinase. Total protein levels were assessed by immuno-blotting using antibodies recognizing the non-phosphorylated form of each protein. Level of actin as a loading reference was detected using anti-β-actin antibodies. The blots were developed with the ECL chemiluminescence system (Amersham Biosciences, Piscataway, NJ, USA). Western blot bands were quantified using Image J software (http://rsbweb.nih.gov/ij/). Antibodies and other reagents used in the current study are listed in the supplemental material.

### Analysis of pre-BCR-induced transcription factor activation by ELISA

Activation of p50 NF-κB1, c-Fos, JunB, NFATc1, Stat1, Stat3 and Stat5 was analysed using the ELISA-based TransAM™ Kit (Active Motif, Carlsbad, CA, USA) and un-stimulated and pre-BCR-stimulated cells. Immobilised oligonucleotide containing a consensus binding site specific of each transcription factor, was incubated with cytoplasmic and nuclear extracts. Bound complex was detected using specific antibody followed by incubation with a secondary antibody conjugated to horseradish peroxidase. Bound material was revealed using a colorimetric readout and results were expressed as optical density (OD).

### Isolation of primary precursor-B lymphocytes

Adult human bone marrow mononuclear cells (ABM009F) were purchased from StemCell Technologies SARL, Grenoble, France. Surface CD19^+^, CD34^lo^ and κ^−^/λ^−^ cells were sorted within the lymphocyte cell gate (Fig. S1b). At least 85% of cells co-expressed surface Vpre-B and µHC, indicating a high enrichment in pre-B II cells (Fig. S1C). The sorted cells were cultured in RPMI 1640 supplemented with 10% (vol/vol) fetal calf serum (FCS), 100 µg/ml Penicillin and 100 µg/ml streptomycin at 37°C in 5% CO_2_.

### Immunofluorescence staining of primary normal pre-B lymphocytes

Cells were treated with anti-μ or control F(ab)'2 antibodies for various length of time, fixed with 4% paraformaldehyde, permeabilised in ice cold ethanol or methanol, and treated with blocking buffer (PBS/0.1% Tween 20/ 2% FCS) before immunostaining. Cells were incubated for 30 min at room temperature with Alexa Fluor 488®-conjugated anti-NFκB p50 or isotype matched antibody as a control. For cellular localization of c-Fos, IRF4 and FoxO3A were first stained with mouse anti-c-Fos, rabbit anti-IRF4 or rabbit anti-FoxO3A for 30 min followed by respective secondary IgG-Ax488. Following extensive washes in (PBS/ 0.1% Tween 20), slides were mounted with Vectashield® containing DAPI for nuclei staining (Vector Laboratories, Burlingame, CA, USA). The staining was visualised (63× Neofluor) using Zeiss Imager M2 Apotome microscope. The ratio for the nuclear verses cytoplasmic protein was determined using ImageJ software (http://rsbweb.nih.gov/ij/).

### Quantitative real time PCR

Total RNA was isolated according to the manufacturer's instructions using RNA extraction kit (RNeasy Kit, Qiagen, Qiagen GmbH, Hilden, Germany). cDNA was synthesised using High capacity cDNA reverse transcription kit (Applied Biosystems, Foster City, CA, USA). Relative quantification of RNA was done by Real Time-PCR Taqman® Gene Expression Assays for *Rag1* (Hs00172121_m1), *Rag2* (Hs00179177_m1), *Pax5* (Hs00172001_m1), *E2A* (Hs00413032_m1), EBF-1 (HS 00395524_m1) obtained from Applied Biosystems. Real time PCR was performed using universal PCR Master Mix (Applied Biosystems) and quantified by ABI PRISM 7900 (Applied Biosystems). Data were normalised with expression of the housekeeping gene GAPDH (Hs99999905_ml) or 18S (Hs99999901_s1) Applied Biosystems), in the PCR reactions and results were expressed as mRNA level (arbitrary units) or fold change.

### Statistical analysis

Statistical analysis was performed using GraphPad Prism (GraphPad Software, La Jolla, CA, USA). Data were analysed by paired Student's *t*-test to compare the gene expression between two groups. Immunofluorescence data were analysed using unpaired Student's *t*-test. Data are presented if not indicated elsewhere as mean ± SD, and was considered to be statistically significant (*P ≤ 0.05) or highly significant (**P ≤ 0.01).

## SUPPORTING INFORMATION

**Figure S1.** Phenotype of pre-B cell lines and sorting method for isolating precursor B cells from human bone marrow mononuclear cells. (A) 697 and Nalm6 cell lines were quantified for their surface markers (VpreB, IgM, Igβ, CD19, Igκ and Igλ) to determine their purity. (B) Mononuclear cells from human bone marrow were stained with anti-CD19 APC, -CD34 PE-Cy7, and -κ/-λ FITC. (C) The precursor B cells (CD19^+^, CD34^lo^, κ^−^ and λ^−^) were sorted within the lymphocyte gate using FACS Aria II sorter. Sorted cells were quantified for surface VpreB and µHC expression. Filled peak represents VpreB^+^ orµHC cell population. **Figure S2.** Pre-BCR-dependent cell cycle entry and proliferation. Six hundred and ninety seven cells were treated with anti-µHC F(ab')_2_ or control F(ab')_2_ antibodies in presence of 3% FCS. Cell cycle analysis showed increased number of cells in S-phase. (A) Trypan blue based cell count showed increased cell growth in pre-BCR stimulated cells compared to control cells. (B) Stimulation of pre-BCR on serum starved cells resulted in phosphorylation of p21^Cip1^ in 2 min and increase in c-Myc level after 24 h. (C) Serum starved 697 cells were treated with anti-µHC or/and anti-CD19 for 15 min or 48 h. The cell lysates were resolved on SDS–PAGE and blotted with antibodies against phospho-Rb, p27^Kip1^ (D), phospho-AKT (E) or phospho-ERK1/2 (F). Actin detection was used as a loading control. The blots represent one of two independent experiments. **Figure S3.** Pre-BCR associated signalosome. Serum deprived 697 or Nalm6 cells were incubated with anti-µHC or control F(ab')2 antibodies or only medium (med) for various time points as indicated (top rows). Phosphorylated forms of various signalling molecules were detected using western blot after pre-BCR crosslinking. (A) LYN and VAV was immunoprecipitated using respective specific antibodies and blotted using anti-phospotyrosine (anti-p-Tyr; 4G10) antibody. BLK, PLCγ-2 and LAB were immunoprecipitated using anti-p-Tyr and blotted using respective specific antibodies. To detect phosphorylated form of SYK, total cell lysate was blotted using phospho-specific antibody. BLNK was immunoprecipitated from the cell lysate and blotted using anti-p-BLNK antibody. (B) Total cell lysates were blotted using respective antibodies for phosphorylated ZAP70, LAT and SLP76. (C) Total cell lysates were blotted using anti-p-AKT, -GSK-3β and -FKHRL1 antibodies. (D) Pre-BCR-induced Ras activation was analysed using pull-down experiments as described in Material and Methods Section. Total lysates of 697 was blotted for ERK and p38 using respective phosphospecific antibodies. Non-phosphorylated forms of all signalling molecules were also detected by re-probing with respective antibodies as a loading control. The blots shown represent one of two independent experiments. **Figure S4.** Pre-BCR-induced activation of c-Cbl and its interaction with Zap70. (A and B) After the stimulation of pre-BCR by anti-µHC F(ab')2 antibodies, the cell lysates (697 or Nalm6) were blotted for detecting c-Cbl (Y774 and Y731). (C) 697 cell lysates were also submitted to immunoprecipitation with anti-ZAP70 Abs. Precipitates were resolved on SDS–PAGE and analysed by western blot for expression of c-Cbl and ZAP70. Data represented is one of two independent experiments. Actin detection was used as a loading control.
