# Supplementary material for: Human pre-B cell receptor signal transduction: evidence for distinct roles of PI3kinase and MAP-kinase signalling pathways
Source: Immun Inflamm Dis. 2013 Oct 30;1(1):26–36. doi: 10.1002/iid3.4 (PMC4217539; doi:10.1002/iid3.4)
Supplement: Supplementary file 1 [file iid30001-0026-SD1.docx]

**Supplemental Information**

**Title: Human Pre-B Cell Receptor Signal Transduction: Evidence for Distinct Roles of PI3-Kinase and MAP-Kinase Signaling Pathways**

**Anbazhagan *et al***

**Supplemental Materials and Methods**

# Reagents

Aprotinin, bestatin, antipain, pepstatin, leupeptin, *N*-octylglucopyranoside and sodium orthovanadate were purchased from Sigma (St Quentin Fallavier, France). The following kinase inhibitors were used: The Src inhibitors - PP1 and the PP2 Pyrazolo-pyrimidine and the inactive analogue PP3 [Calbiochem*-*Novabiochem Co*,* San Diego, USA]; SYK kinase inhibitor - BAY 61-3606 [Coger SA, Paris, France], PI3K inhibitor - LY294002 (Sigma, St Quentin Fallavier, France); ERK inhibitor - PD88059 and MEK1/2 inhibitor - U0126 [LC Labs, Woburn, USA]

**Antibodies**

The following antibodies were used for western blots: antibodies directed to Blk, [Abcam, Paris, France]; GSK3 α/β [Affinity BioReagents Inc., Rockford, USA], BLNK, Btk, c-Cbl, LAT, SLP76, Rag1, phospho (p)-c-Cbl (Tyr 731), p-c-Cbl (Tyr 774), p-LAT, p-SLP76, [Becton Dickinson Transduction Laboratories, Lexington, USA]; ERK (p44/42 MAPK), IkB-α, NF-κB (p105/50), p38 MAPK, p-AKT (Ser473), p-BLNK (Tyr96), p-Btk (Tyr223), p-Erk (p-p44/42 MAPK), p-GSK3α (Ser9)/β (Ser21), p-IκBα (Ser32/36), p-LYN (Tyr507), p-p38 (Thr180/Tyr182), p-PLCγ2 (Tyr1217), p-SYK (Tyr525/526), p-ZAP70 (Tyr319)/p-SYK (Tyr352), p-ZAP70 (Tyr493), p-IKKα/IKKβ (Ser181), IKKα, ZAP70, LAB [Cell Signaling Technology, Boston, USA] ; β-Actin, AKT (H-136), FKHRL1 (N-16), LYN, PLC-γ2, SYK, Vav, K-Ras, JNK, c-Fos [Santa Cruz Biotechnology, CA, USA], p-FKHRL1 (Ser253), p-Tyr 4G10 (mouse), Goat IgG F(ab')2 fragments anti-human μHC (Fc5µ) were used for pre-BCR stimulation and goat IgG F(ab')2 without defined specificity as control [Jackson ImmunoResearch Laboratories, Suffolk, UK]**.** Antibodies used for cell cycle related and other proteins were as follows: p21^Cip1^, p27^Kip1^, p-p21^Cip1^ (Ser146) [Santa Cruz Biotechnology Inc., USA], c-Myc, Rb [BD transduction laboratory, Lexington, USA], p-Rb (Ser807/811). Antibodies for flow cytometry analysis and cell sorting were as follows: CD179a-R-phycoerythrin [Biolegend, San Diego, USA], μHC-R-phycoerythrin, κ LC and λ LC-fluorescein isothiocyanate, CD34- phycoerythrin cyanine-7 and CD19-allophycocyanin [Becton Dickinson] were used. Antibodies used for immunofluorescence microscopy include: AlexaFluor488-conjugated anti-NFκB p50, anti-cFos antibody [Biolegend, San Diego, USA], rabbit anti-IRF4 antibody [Cell Signaling Technology, Boston, USA] and rabbit anti-FoxO3A antibody [Millipore, USA]. Antibodies used against cell cycle related and other proteins were as follows: p21^Cip1^, p27^Kip1^, p-p21^Cip1^ (Ser146) [Santa Cruz Biotechnology Inc., USA], c-Myc, Rb [BD transduction laboratory, Lexington, USA], p-Rb (Ser807/811). We used anti-IRF4 antibody [Cell Signaling Technology, Boston, USA] for immunofluorescence studies.

## Immunoprecipitation and pull-down assays

Cell lysates were subjected to immunoprecipitation with appropriate antibodies and 50 µl of protein G sepharose (Amersham Biosciences). Eluted proteins were then analysed by western blot using specific antibodies. Pull-down assays were performed as previously described [51], after respective treatments of serum starved cells.

**Analysis of pre-BCR induced cell cycle and cell proliferation**

For cell cycle analysis, cells (1 x10^5^) were cultured overnight in serum free RPMI medium, then stimulated with F(ab’)2 anti-μHC (or control) in medium containing 3 % FCS for indicated time. After stimulation, cells were washed in PBS containing 5 mM EDTA and resuspended in 300 μl of the same buffer. Cells were then fixed in 700 μl of 100 % ethanol and centrifuged at 400 x g. The pellet was resuspended in 500 μl of PBS-EDTA and incubated at room temperature for 30 min in the presence of 50 μl RNaseA (10 mg/ml) followed by addition of 500 μl propidium iodide (100 μg/ml in PBS). DNA content was measured using the Cychred software (University of Wales College of Medicine, Cardiff, Wales). To examine the effects of pre-BCR ligation on cell proliferation, 697 cells were cultured overnight in serum-free culture medium, prior to stimulation with anti-μHC F(ab’)2 or control F(ab’)2 antibodies in medium containing 2 % FCS. Cells were enumerated every 24 h using trypan blue exclusion method.

**Supplemental Figures**

**
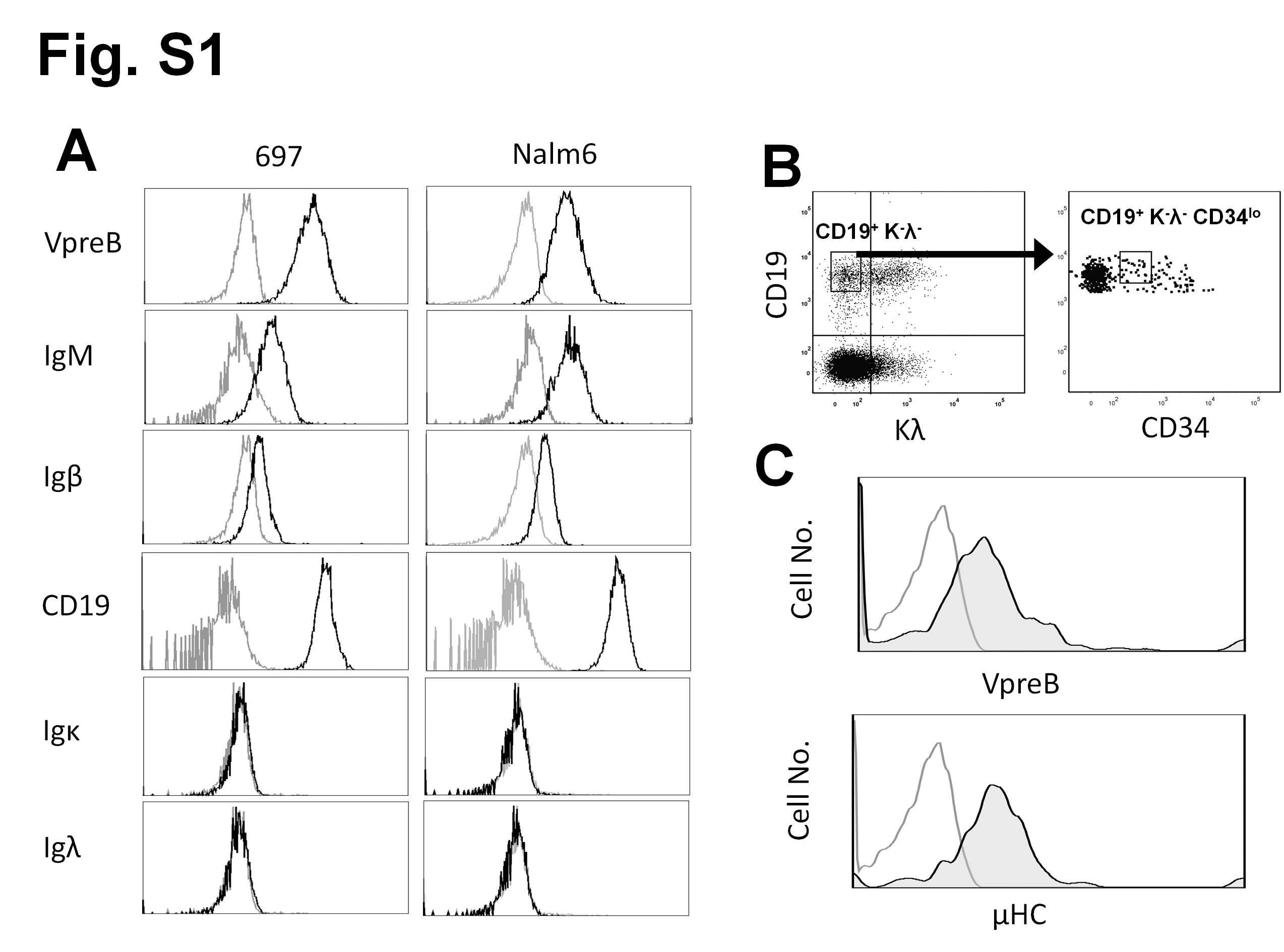
**

**Figure S1: Phenotype of pre-B cell lines and sorting method for isolating precursor B cells from human bone marrow mononuclear cells.**

**(a)** 697 and Nalm6 cell lines were quantified for their surface markers (VpreB, IgM, Igβ, CD19, Igκ and Igλ) to determine their purity. **(b)** Mononuclear cells from human bone marrow were stained with anti-CD19 APC, -CD34 PE-Cy7, and -κ/-λ FITC. **(c)** The precursor B cells (CD19^+^, CD34^lo^, κ^-^ and λ^-^) were sorted within the lymphocyte gate using FACS Aria II sorter. Sorted cells were quantified for surface VpreB and µHC expression. Filled peak represents VpreB^+^ or µHC cell population.

**
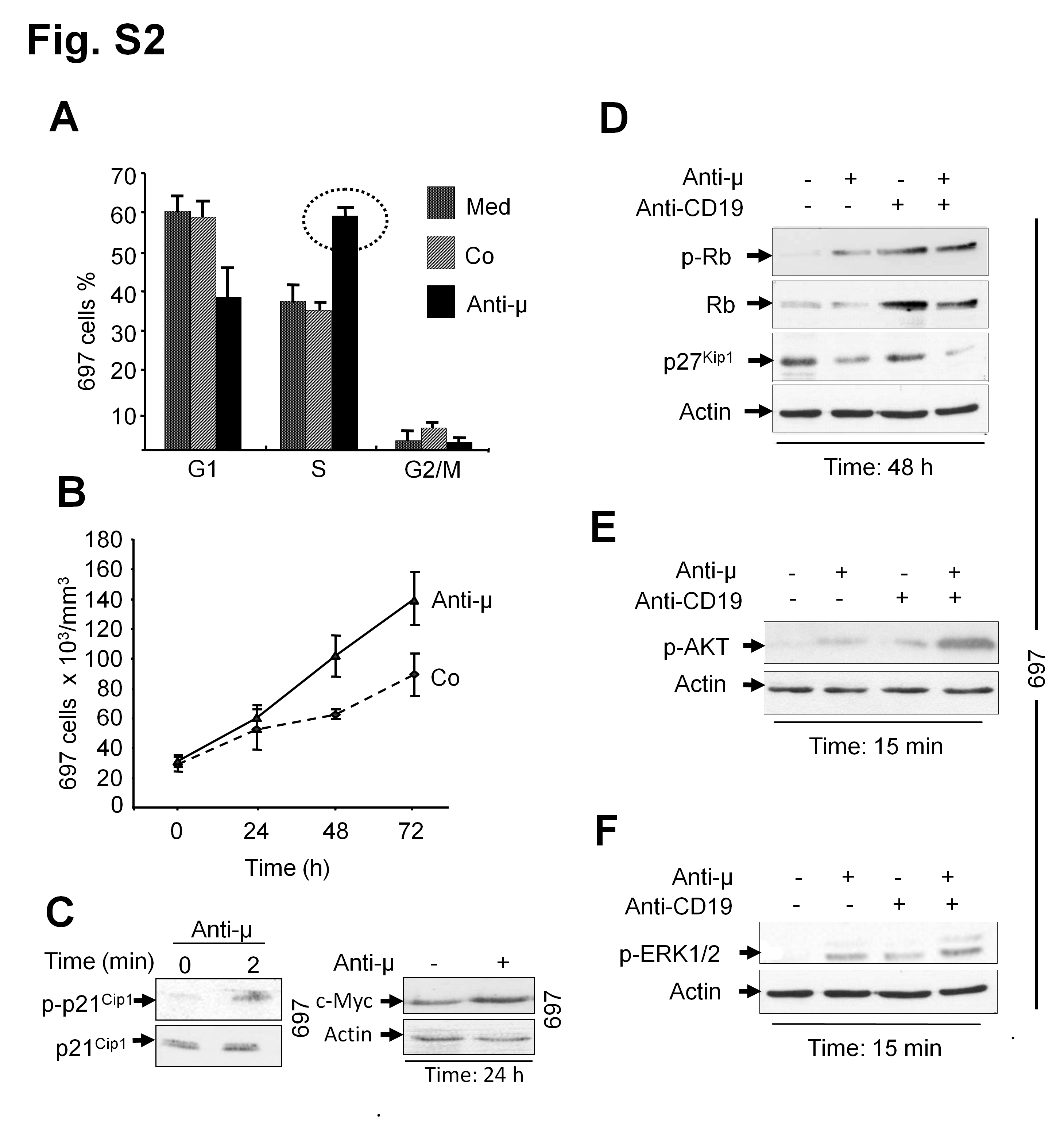
**

**Figure S2: Pre-BCR-dependent cell cycle entry and proliferation.**

697 cells were treated with anti-µHC F(ab’)_2_ or control F(ab’)_2_ antibodies in presence of 3 % FCS. Cell cycle analysis showed increased number of cells in S-phase **(a).** Trypan blue based cell count showed increased cell growth in pre-BCR stimulated cells compared to control cells **(b).** Stimulation of pre-BCR on serum starved cells resulted in phosphorylation of p21^Cip1^ in 2 min and increase in c-Myc level after 24 h **(c)**. Serum starved 697 cells were treated with anti-µHC or/and anti-CD19 for 15 min or 48 h. The cell lysates were resolved on SDS-PAGE and blotted with antibodies against phospho-Rb, p27^Kip1^ **(d),** phospho-AKT **(e)** or phospho-ERK1/2 **(f)**. Actin detection was used as a loading control. The blots represent one of two independent experiments.

**
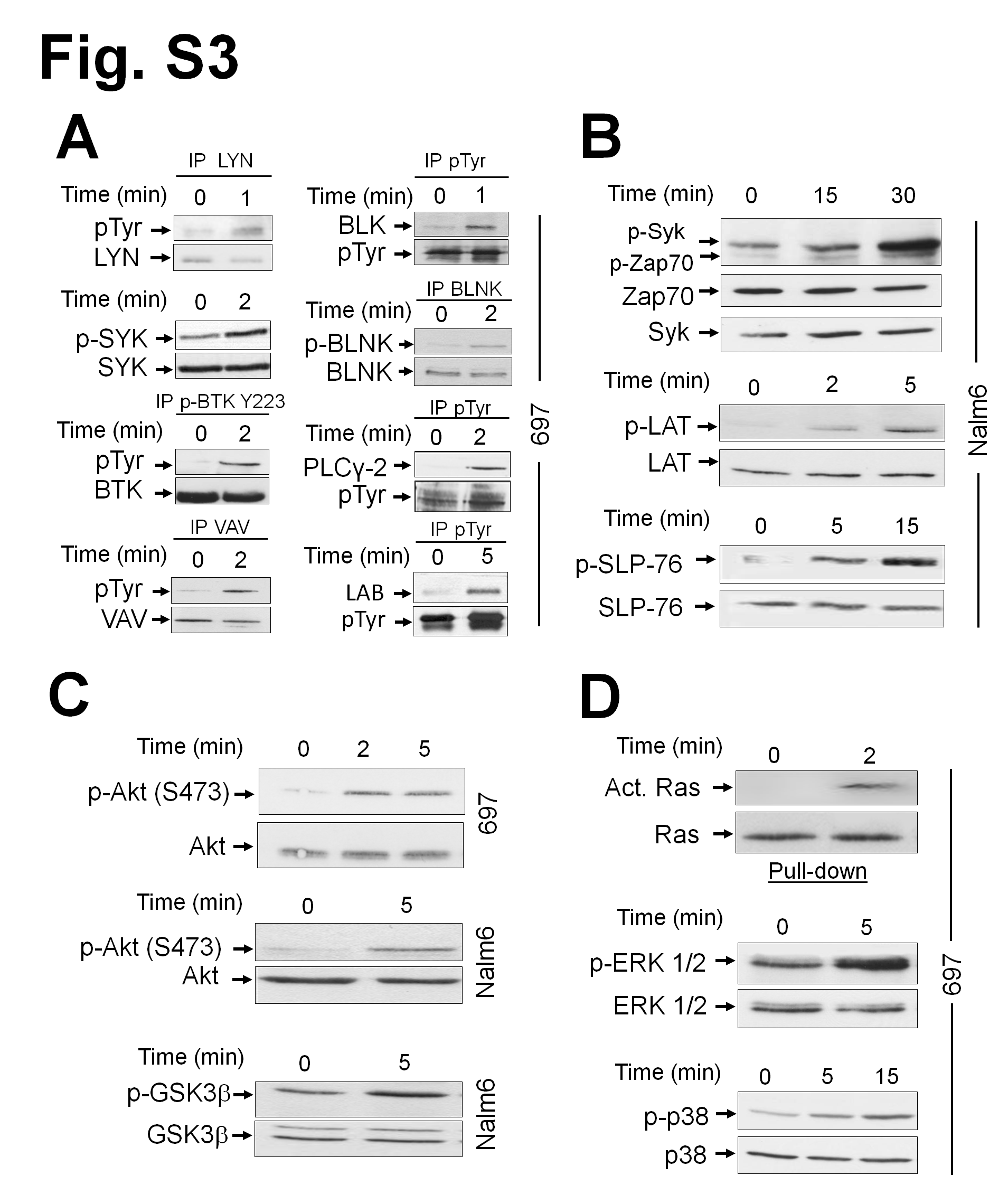
**

**Figure S3: Pre-BCR associated signalosome**

Serum deprived 697 or Nalm6 cells were incubated with anti-μHC or control F(ab’)2 antibodies or only medium (Med) for various time points as indicated (Top rows). Phosphorylated forms of various signaling molecules were detected using western blot after pre-BCR crosslinking. **(a)** LYN and VAV was immunoprecipitated using respective specific antibodies and blotted using anti-phospotyrosine (anti-p-Tyr; 4G10) antibody. BLK, PLCγ-2 and LAB were immunoprecipitated using anti-p-Tyr and blotted using respective specific antibodies. To detect phosphorylated form of SYK, total cell lysate was blotted using phospho-specific antibody. BLNK was immunoprecipitated from the cell lysate and blotted using anti-p-BLNK antibody. **(b)** Total cell lysates were blotted using respective antibodies for phosphorylated ZAP70, LAT and SLP76. **(c)** Total cell lysates were blotted using anti-p-AKT, -GSK-3β and -FKHRL1 antibodies. **(d)** Pre-BCR-induced Ras activation was analysed using pull-down experiments as described in Material and Methods. Total lysates of 697 was blotted for ERK and p38 using respective phosphospecific antibodies. Non-phosphorylated forms of all signaling molecules were also detected by re-probing with respective antibodies as a loading control. The blots shown represent one of two independent experiments.

**
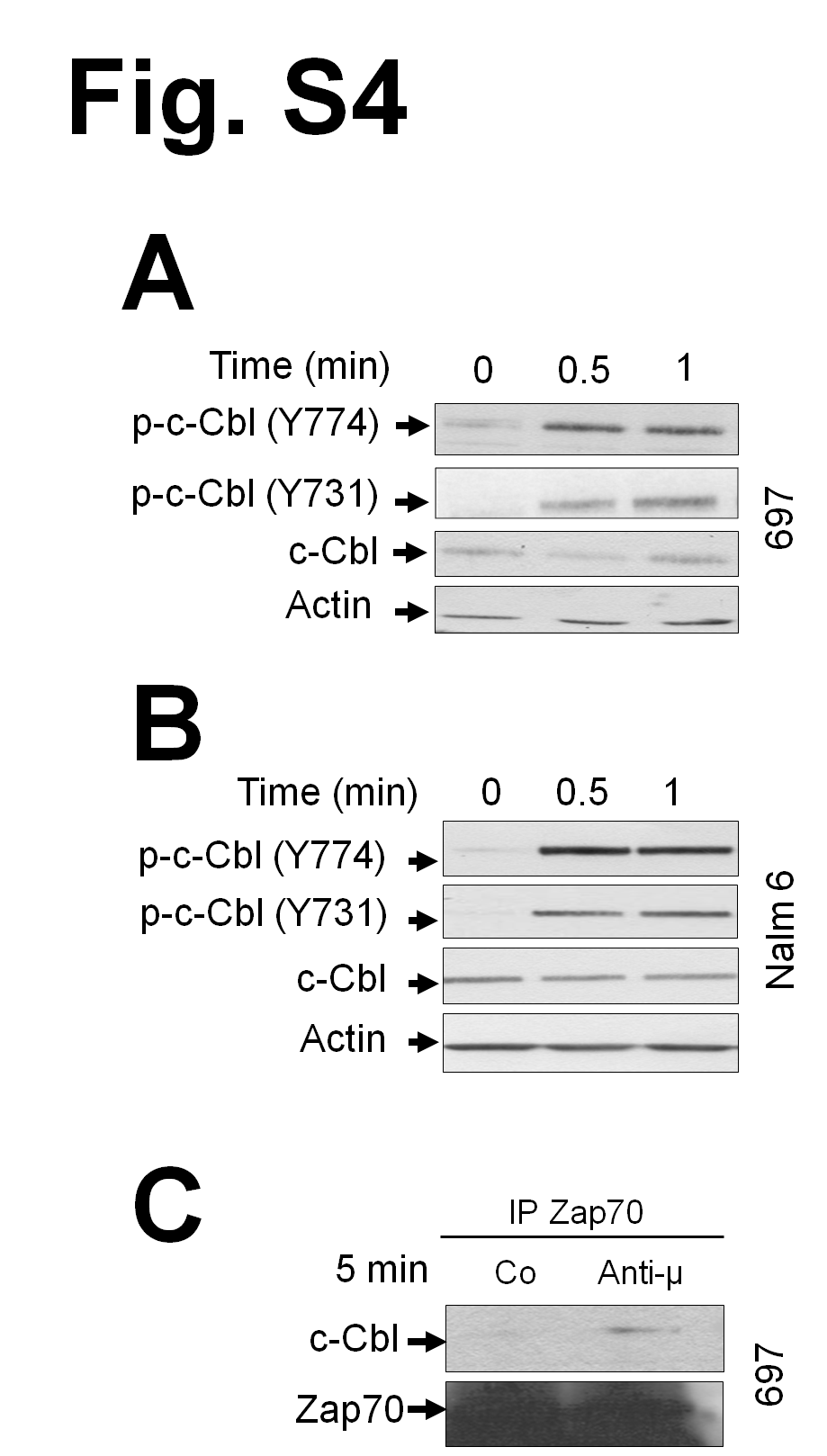
**

**Figure S4: Pre-BCR-induced activation of c-Cbl and its interaction with Zap70.**

**(A and B)** After the stimulation of pre-BCR by anti-μHC F(ab’)2 antibodies, the cell lysates (697 or Nalm6) were blotted for detecting c-Cbl (Y774 and Y731). **(C)** 697 cell lysates were also submitted to immunoprecipitation with anti-ZAP70 Abs. Precipitates were resolved on SDS-PAGE and analyzed by western blot for expression of c-Cbl and ZAP70. Data represented is one of two independent experiments. Actin detection was used as a loading control.
